# Identification of Parathyroid Glands Through Near-Infrared Autofluorescence During Thyroid Surgery: Retrospective Analysis of the Impact on Post-Operative Hypocalcemia Rate Reduction and Potential Improvement of Healthcare Expenditure

**DOI:** 10.3390/medicina61111985

**Published:** 2025-11-05

**Authors:** Davide Inversini, Matteo Zanchetta, Niccolò Enrico Perego, Andrea Palillo, Caterina Franchi, Enrico Ferri, Giuseppe Ietto, Giulio Carcano

**Affiliations:** 1 General, Emergency and Transplant Surgery Department, ASST Settelaghi, University of Insubria, 21100 Varese, Italy; 2 Department of Medicine and Technological Innovation (DiMIT), University of Insubria, 21100 Varese, Italy; 3 Unit of General Surgery and Surgical Oncology, Department of Medicine, Surgery and Neurosciences, University of Siena, 53100 Siena, Italy; 4 Medicine and Surgery, University of Insubria, 21100 Varese, Italy

**Keywords:** parathyroid glands, post-operative hypocalcemia, thyroid surgery, near-infrared autofluorescence, NIRAF, health care spending, disease burden

## Abstract

*Background and objectives*: The identification and preservation of the parathyroid glands (PGs) are of paramount importance in thyroid surgery. Permanent hypoparathyroidism represents a significant post-operative complication that may result from the surgeon’s failure to accurately identify the PGs or their associated blood supply. Post-operative hypocalcemia (POH) represents the most common surgical complication following thyroid surgery and is the primary factor influencing the post-operative course, causing the requirement of frequent electrolyte monitoring and of supportive therapies, precluding the possibility of early discharge, and resulting also in significant healthcare costs. Near-infrared autofluorescence (NIRAF) employs the intrinsic capacity of PGs to emit fluorescent light when exposed to light within the NIR spectrum. In the context of thyroid surgery, NIRAF represents a safe method with no documented risks to the patient. *Materials and Methods*: A retrospective analysis was conducted on 383 patients who underwent thyroid surgery at our Institution, either with (n = 27) or without (n = 356) intraoperative NIRAF, focusing on its efficacy in reducing the incidence of POH. *Conclusions*: Our results suggest that the systematic integration of NIRAF within non-high-capacity centres for cervical surgery, owing to its usefulness in identifying PGs intraoperatively, may strongly contribute to a reduction in the incidence of POH (45.8% without vs. 18.5% with NIRAF; *p* = 0.0078). This, in turn, has the potential to contribute to a decrease in overall healthcare expenditure.

## 1. Introduction

The identification and preservation of the parathyroid glands (PGs) are of paramount importance in thyroid surgery. Permanent hypoparathyroidism represents a significant postoperative complication that may result from the surgeon’s failure to accurately identify the PGs or their associated blood supply [1,2]. Post-operative hypocalcemia represents one of the most common surgical complications following thyroid surgery, with the reported incidence of transient hypocalcemia ranging from less than 1% to approximately 50% according to single-centre, large national studies, and a systematic review, and it is the primary factor influencing the post-operative course [1,3,4,5,6]. In patients with hypocalcemia, the necessity for frequent electrolyte monitoring and the administration of intravenous or oral supportive therapies for the management of symptoms precludes the possibility of early discharge prolonging the length of hospital stay (LHS), resulting also in significant healthcare costs [7]. Autofluorescence (AF) is the emission of light produced naturally by intrinsic fluorophores, which are photoreactive chemicals that absorb and emit energy in a predictable manner, whereby light excitation results in re-emission of light. This phenomenon occurs when the intrinsic fluorophores are illuminated by higher-energy light (shorter wavelengths) and subsequently emit lower-energy light (longer wavelengths) that can be detected by an identification system. The term AF is used to distinguish this process from the emission of light which instead results from the administration of exogenous fluorophores (e.g., methylene blue and indocyanine green). Near-infrared autofluorescence (NIRAF) employs the intrinsic capacity of parathyroid tissue to emit fluorescent light when exposed to light within the near-infrared spectrum [8]. The methodical implementation of this technology within high-capacity centres for cervical surgery could have a considerable impact on reducing the occurrence of POH and consequently the LHS, therefore potentially contributing to a reduction in the overall health care expenditure [9]. Considering its ease of use and learning curve, non-invasiveness, and safety, allowing also less experienced surgeons to better identify the tissues [10,11], it can be similarly postulated that in the future the same positive outcomes could benefit also non-high-capacity centres for cervical surgery where procedures performed by less experienced surgeons carry a higher risk of complications including POH [12].

The aim of this research was to assess the effectiveness of NIRAF in correctly detecting PGs during thyroid surgery and consequently reduce the occurrence of POH. A secondary endpoint of the study was to investigate the potential existence of any correlations between pre-operative characteristics, POH, postoperative complications, and LHS. It is hypothesized that a decrease in the incidence of POH and the resulting reduction in LHS would be indicative of a contribution by the systematic implementation of NIRAF to a decrease in healthcare expenditure.

## 2. Materials and Methods

**Figure 1:**
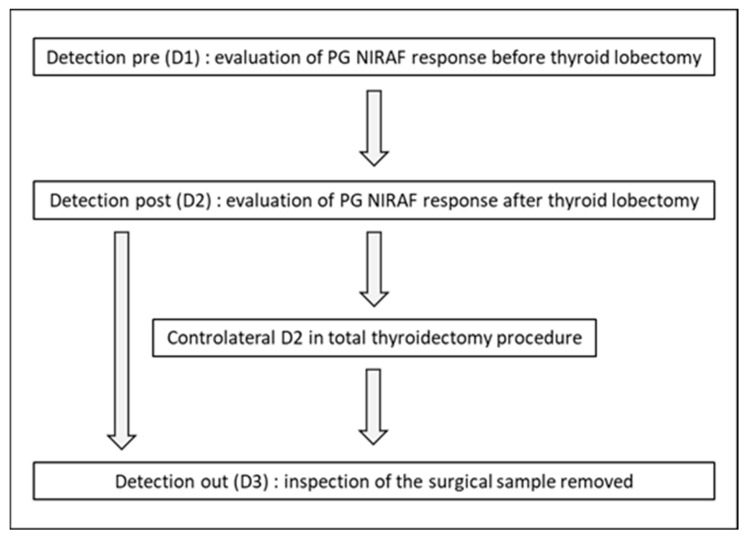
Analysis protocol. We designed the protocol.

A retrospective observational analysis was conducted of a time window commencing on the 1 January 2018 and concluding on the 31 December 2022, encompassing all consecutive adult patients who underwent either total thyroidectomy or hemithyroidectomy in the Emergency Surgery and Transplantation Department at Circolo Hospital in Varese, Italy. From the 1 May 2022 to the 30 June 2022, NIRAF was utilized to identify PGs during thyroid surgery. The NIRAF technology is based on the ability of parathyroid tissue to emit fluorescent light when irradiated with light at a wavelength of 785 nm, i.e., near-infrared light. The instrument, consisting of a laser source and a camera with a fluorescence sensor, was provided by Medtronic (Milano, MI, Italy) and is called IR EleVision. The NIRAF camera was used in every procedure carried out at our centre during the reporting period. Intraoperative neuromonitoring (IONM) of the recurrent laryngeal nerve (RLN) was always performed during thyroid surgery. According to a protocol that we designed, for each considered patient we evaluated the PGs NIRAF response both before the thyroid dissection (D1, detection pre-thyroidectomy), and in the thyroid bed and surrounding area after the resection (D2, detection post-). The surgical thyroid sample was subsequently inspected with NIRAF for the accidental removal of the PGs (D3, detection out). In cases of total thyroidectomy, both thyroid lobes were subjected to the same examination. Figure 1 provides an overview of the methodology employed.

Patient clinical characteristics and demographics, results of PG NIRAF identification, measurements of fluorescence intensity, and records of the postoperative complications were systematically gathered and catalogued in a database. Subsequently, the data were retrospectively compared with the data of patients who underwent total thyroidectomy or hemithyroidectomy without the use of NIRAF in our Institution during the time frame considered. The patients were divided into two groups: Group 1 ‘with fluorescence’ where PGs were identified intraoperatively using NIRAF, and Group 2, ‘without fluorescence’ where PGs identification was conducted using conventional techniques. Inclusion criteria were patients aged ≥18 years, undergoing elective total thyroidectomy or hemithyroidectomy procedure, with full capability to understand Italian. Exclusion criteria for the study were renal and calcium excretion pathologies, thyroidectomy with planned therapeutic or prophylactic lymphadenectomy, and indication for simultaneous parathyroidectomy. In accordance with the literature, a calcemic value below 8.5 mg/dL persisting for more than six months after surgery was deemed as diagnostic for permanent hypocalcemia [13,14,15]. The primary endpoint was the identification of temporary and permanent POH in Groups 1 and 2. The secondary endpoint was to investigate any possible correlation between preoperative characteristics and POH, as well as the LHS (expressed in days +/− standard deviation (SD). Additionally, the study evaluated the correlation between the incidence of complications and postoperative LHS. Major complications were defined as loss of signal (LOS) of the IONM, POH, hemorrhage requiring reintervention, and dysphonia. Patients were classified as ‘complicated’ if they experienced at least one major complication, and ‘uncomplicated’ if they experienced none. In cases where the IONM signal was lost, the surgeon opted for a two-stage thyroidectomy to prevent potential damage to both RLNs. During the follow-up (FU) period, serum calcemic levels were assessed at 24 h, 48 h, one month, and six months post-surgery. A postoperative laryngoscopy was performed within 48 h of surgery, and parathormone (PTH) levels were measured within one month of the surgical procedure. The statistical analyses were carried out using Lumivero XLSTAT. For all categorical variables (e.g., sex, presence or absence of LOS), Fisher’s exact test (TEF) was utilized. For all continuous variables (e.g., age, days of hospitalization), Student’s *t*-test (TS) was utilized. The level of statistical significance was established at *p* < 0.05. All patients consented to the utilization of their data and provided their signature on a written informed consent form. We obtained approval from the Institutional Review Board (IRB) of Ospedale di Circolo e Fondazione Macchi for this clinical study (registration number n° 0119180).

## 3. Results

A total of 383 patients underwent thyroid surgery (hemithyroidectomy or total thyroidectomy) at our Institution during the specified time period. There were 27 patients in Group 1, and 356 patients in Group 2. Table 1 presents data regarding patient characteristics, preoperative diagnoses, type of surgical procedure and postoperative diagnoses. The two groups exhibited uniform characteristics, with the exception of the preoperative diagnosis, which demonstrated a direct correlation with the high variability of cytology in relation to the American Bethesda Classification [16].

During all the procedures conducted with NIRAF, at least one PG was identified.

In the course of total thyroidectomy procedures utilizing NIRAF (n = 19), 74 (97.4%) out of 76 PGs were successfully identified intraoperatively. In two separate procedures, it was not possible to identify all four PGs intraoperatively, as only three images were compatible with parathyroid structures. In both cases, the two unidentified glands were superior PG.

During the hemithyroidectomy procedures with NIRAF (n = 8), 31 (96.8%) PGs were positively identified. The one gland that could not be identified was an inferior one. However, during the procedure, two images observed at two distinct locations in the lower pole were plausibly consistent with parathyroid tissue.

Considering the three cases where NIRAF was unable to correctly detect a PG, the surgeon was nevertheless able to discern structures that exhibited characteristics suggestive of parathyroid tissue through visual examination. In one of the aforementioned cases, two sites were identified by NIRAF as compatible with parathyroid tissue. Two of these cases occurred during the first week of the study, while the third occurred during the third week.

The D1 examination was identical to D2, confirming that the PG were accurately preserved after the thyroid lobectomy. At D3, no images compatible with parathyroid tissue were identified. Final histological examinations corroborated this result.

In two instances, an elevated fluorescence intensity was observed in other regions of the surgical field after the identification of four PGs, leading to the documentation of potential supernumerary ectopic parathyroid tissue. However, due to the atypical location and intensity of fluorescence, it was not possible to definitively ascertain whether these findings were false positives. Table 2 displays the comparison of PG non-identification and accidental removal between Group 1 and Group 2.

There were no statistically significant differences found in the analysis of the number of PG that were not identified during surgery and in the incidence of accidental removal of PG between the two groups. However, 20 cases (5.6%) of accidental removal of parathyroid tissue (confirmed by definitive histological examination) were documented in Group 2, whereas no cases of accidental removal of parathyroid tissue occurred in Group 1.

Table 3 presents the data on postoperative major complications. Transient POH was observed in 163 cases (45.8%) of Group 2 and in only 5 cases (18.5%) of Group 1. This difference was statistically significant (*p* = 0.0078). Group 2 experienced permanent hypocalcemia in 4 cases (1.1%), whereas no cases of permanent hypocalcemia were reported in Group 1. In Group 2, LOS occurred in 21 cases (5.8%), with only one case (0.3%) of subsequent permanent chordal paralysis. In Group 2, 12 cases (3.4%) necessitated re-intervention due to hemorrhage.

Due to the increased risk of developing POH after total thyroidectomy, this study compared postoperative complications between subgroups undergoing hemithyroidectomy with fluorescence and hemithyroidectomy without fluorescence (Table 4), as well as total thyroidectomy with fluorescence and total thyroidectomy without fluorescence (Table 5). Transient POH occurred significantly more after total thyroidectomies performed without NIRAF (52.8%) than after those performed with NIRAF. No statistically significant difference was observed between the hemithyroidectomy subgroups, although transient POH occurred in 12 cases (8.4%) in the absence of NIRAF, and never with NIRAF.

The correlation between postoperative major complications and LHS was analysed. Table 6, Table 7 and Table 8 present the data for comparison.

The data demonstrates that POH and hemorrhage have the most significant impact on the duration of post-operative hospitalization. The presence of POH was found to significantly prolong the LHS for all patients (*p* = 0.0012), Group 1 (*p* < 0.0001), and Group 2 (*p* = 0.0049). Postoperative hypocalcemia extended the hospitalization time for complicated patients (5.38 ± 5.56 days) compared to uncomplicated patients (3.94 ± 2.91 days) with statistical significance (*p* = 0.0012). The analysis indicates that POH is a significant contributor to prolonged hospital stay both in Group 1 (7 ± 2.96 vs. 3.6 ± 0.5 days, *p* < 0.0001), and Group 2 (5.31 ± 5.61 vs. 3.99 ± 3.07 days, *p* = 0.0049) patients. In Group 2, hemorrhage requiring reintervention was shown to be a significantly influential factor (*p* < 0.0001) in extending hospital stay after the surgery, with an average of 13.66 ± 17.47 days as opposed to 4.28 ± 2.75 days in patients without complications. Our data shows that POH is the primary factor responsible for the prolongation of the postoperative hospitalization period. The importance of this figure is further highlighted in the NIRAF group, where the *p*-value is less than 0.0001.

The impact of NIRAF on both operating time and LHS was investigated. Table 9 and Table 10 present the data for these two variables, categorized based on the type of thyroid surgery.

There were no statistically significant differences in the duration of surgery between procedures with and without NIRAF. Hemithyroidectomies with NIRAF were slightly longer (65 ± 15 min) than those without NIRAF (57.91 ± 21.94 min) (*p* = 0.3778). On the opposite, total thyroidectomies with NIRAF were slightly shorter (79.47 ± 8.13 min) than those without NIRAF (86.10 ± 28.35 min) (*p* value = 0.3111).

Likewise, the postoperative hospitalization duration did not show statistically significant difference among the groups examined.

The potential correlation between patients’ individual characteristics and complications was studied (Table 11).

No predictive variables were found for the development of complications in the compared groups.

Data regarding FU through measurement of serum values of calcium and PTH were collected (Table 12).

Group 1 had higher levels of serum calcium and PTH compared to Group 2. However, statistical significance (*p* = 0.0019) was observed only for calcemia at one month after surgery (9.46 ± 0.65 mg/dL Group 1 vs. 9.12 ± 0.54 mg/dL Group 2; *p* = 0.0019).

## 4. Discussion

Accurate identification of the PG during thyroid surgery is crucial to reduce POH, a common and significant complication. The present study analyses the use of NIRAF to identify PG during thyroid surgery and the potential correlation with several variables. Consistently with literature, our study showed a rate of correct identification of 97.4% for total thyroidectomy procedures, and of 96.8% for hemithyroidectomy procedures. Kim et al. [17] conducted a meta-analysis of 17 studies involving 1198 patients and reported a 96% sensitivity in PG identification. Abbaci et al. [18] conducted a systematic review of 47 scientific papers involving 1615 patients, demonstrating that AF can help identify PG in 76.3% to 100% of patients with a sensitivity and specificity of 94.1% and 80%, respectively, with false positives potentially attributable to brown adipose tissue [19]. During our study, we identified an anomalous region of markedly elevated intensity in two cases after the identification of the four PG. The surgeon conducted a visual inspection of the area of emission and identified characteristics consistent with a nodule of brown adipose tissue, rather than an ectopic parathyroid. However, due to ethical considerations, the specimen was not removed, and therefore there is no histological confirmation of its nature. In the first multicenter study on this subject, Kahramangil et al. reported an AF identification rate of 584 out of 594 PG (98.3%) [20]. Falco et al. [21] conducted a study on 74 patients which demonstrated that the AF method significantly increases the possibility of identifying PG compared to simple visual assessment. As detailed in the meta-analysis conducted by Wang et al. [22], the majority of analysed studies report NIRAF accuracy levels of approximately 100% [23,24,25], while only a minority report document accuracy rates below 85% [26,27]. This heterogeneity of results could be due to differences in the instruments used. As reported by Solorzano [28] and Kim et al. [17], both probe-based and image-based NIR detection devices have advantages and disadvantages that can lead to differences in NIRAF accuracy. Probe-based systems provide real-time auditory feedback when the probe contacts parathyroid tissue, while image-based systems offer spatial localization of the glands without the need for direct tissue contact. However, image-based systems require complete darkness in the operating room, and the intensity of AF may be affected by the distance of the camera from the operating field and is subjectively assessed by the operator.

The international literature agrees on the usefulness of AF in identifying PG and analyses conducted on large case series demonstrate statistically significant differences in favor of NIRAF [17,22,29]. Tjahjono et al. have highlighted the potential utility of NIRAF technology as an adjunct to conventional thyroid surgery, enabling the accurate identification of PG [30]. However, they emphasized the necessity for its utilization to be based on intra-operative direct visualization by the surgeon. Although no statistically significant data emerged, our study indicated that the “NIRAF” group demonstrated superior accuracy in avoiding accidental removal, as no PG were accidentally removed when using AF, in contrast to the 20 PGs accidental removals that occurred in the group that did not utilize NIRAF (5.61%) (Table 2). We believe that this lack of statistical significance probably derives from the small sample size, meaning that a real difference may have been missed. This percentage is lower than the range identified in the literature (8% to 19%) [31], thereby confirming that the accidental removal of PGs during thyroid surgery is low when performed in high-volume centres. Furthermore, the heterogeneity of the studies conducted on AF may be a confounding factor in the analysis of the data regarding the accuracy of the method, as some of the studies included in the meta-analyses employed hybrid techniques for gland detection. Despite these limitations, our analysis is consistent with the majority of published work.

In accordance with the American Association of Clinical Endocrinology, hypocalcemia was defined as a serum calcium value lower than 8.5 mg/dL [13]. The definition did not consider post-operative PTH values or the presence or absence of associated symptoms [31]. The incidence of transient hypocalcemia in the existing literature ranges from 0.3% to 49%, while the incidence of permanent hypocalcemia ranges from 0% to 13%. The incidence is observed to be lower in high-volume centres [32,33,34]. The variability of data is attributable to the disparate definitions of hypocalcemia employed by the authors, which renders comparison between cases a challenging undertaking [31]. Our study found an overall incidence of transient hypocalcemia of 43.86% and permanent hypocalcemia of 1.04%. Despite the broad definition of hypocalcemia employed in this study, the findings are in accordance with those previously documented in the literature. The utilization of NIRAF has been demonstrated to balance out the intrinsic risk factors associated with POH, as evidenced by extensive case series studies [35,36,37,38]. In our case series, the incidence of transient POH was significantly lower following surgery performed with NIRAF (18.5%) compared to surgery with conventional PG identification (45.8%). However, the present study’s data are insufficient to adequately assess the correlation between NIRAF and permanent POH. In a recent prospective study considering patients with primary papillary thyroid carcinoma undergoing total thyroidectomy and bilateral neck dissection, the use of NIRAF allowed for the accurate identification of the upper PG in over 95% of cases and the lower PG in over 85% of cases. This resulted in glandular preservation and a reduced incidence of temporary and symptomatic hypocalcemia compared to the control group. Additionally, PTH levels were normal for all patients who underwent surgery with NIRAF one month after the procedure, whereas one patient in the control group was diagnosed with permanent hypoparathyroidism [39].

A recent meta-analysis revealed an absence of correlation between postoperative hypoparathyroidism and the NIRAF method [40]. Similarly, there was no correlation in our case series. It is noteworthy that the exclusion criteria included unilateral procedures. In the case study presented here, we also evaluated the incidence of hypocalcemia in patients who had undergone hemithyroidectomy. As anticipated, no statistically significant results were obtained. Although it is widely acknowledged in the literature that this complication has a higher incidence in bilateral procedures, it is plausible that NIRAF utilization may also help in reducing hypocalcemia following hemithyroidectomies.

Weng et al. analyzed six studies including 2180 patients and reported that the incidence of hypocalcemia is at least three times higher in cases of total thyroidectomy without NIRAF compared to the same procedure performed with NIRAF [41].

The data pertaining to the prevalence of permanent hypocalcemia in patients undergoing surgical procedures with the assistance of NIRAF is encouraging. Although the results did not reach statistical significance, the use of NIRAF suggests that a reduction in the percentage of hypocalcemia beyond six months after surgery may be possibly achieved. It is possible that even with the analysis of multicentre data, the low incidence of permanent POH may not yield statistically significant evidence of a reduction in complications. It may be worthwhile to consider the possibility that continued utilisation of NIRAF could potentially yield further data that could one day demonstrate significance, even in relation to the permanent component. If we are correct in our interpretation of the findings, it would seem that these considerations provide a useful illustration of the benefits of utilising NIRAF.

Our research has identified a correlation between NIRAF and a reduction in the incidence of hypocalcemia (defined as serum calcium values below 8.5 mg/dL) at 24, 48, one, and six hours, and one, six, and twelve months. It is, however, important to note that the statistically significant finding was limited to the analysis of calcemia at one month. It is important to note that this result is based on the analysis of all patients, not just those with complications. Moreover, the presence of confounding factors, such as the administration of calcium and vitamin D support therapy, which is commonly prescribed following thyroid surgery, may have influenced the results.

The analysis did not identify any risk factors that may predict hypocalcemia. In light of the statistically significant differences in preoperative diagnosis between the two groups, configuring a higher-risk profile for the NIRAF group (i.e., a higher malignancy rate), the lower incidence of POH underscores the potent protective effect of NIRAF technology. In contrast, previous studies have indicated that the diagnosis of malignancy, lymphadenectomy of the central compartment, and re-intervention (i.e., more complex surgery) are risk factors for POH [41].

Falco et al. observed that the recognition of PGs is more challenging during lymph node dissections and surgical revisions due to the presence of numerous adhesions [21]. Similarly, Prete et al. corroborated these findings [42]. The result of surgical procedures in which such risk factors are present is contingent upon the experience of the performing surgeon. It can be postulated that in multicenter studies involving non-high-volume thyroid surgery centres, the incidence of hypocalcemia is more closely associated with cases of ‘difficult thyroidectomy’. Nevertheless, the analysis of data collected exclusively in high-volume centres with highly experienced surgeons may not corroborate this evidence. It can be reasonably deduced that the primary source of bias in many studies is the experience of the operator. A further consideration is the potential role of AF in more complex surgical cases, whereby it may facilitate a more accurate and expedient identification of the parathyroids, thereby reducing the risk of POH. Regarding technical difficulties, we failed to identify parathyroids in the examined sample during the first and third week of the study. This outcome was attributed to the learning curve inherent to the methodology, which requires meticulous preparation and execution. To date, no specific studies have been conducted on the learning of NIRAF [18]. Makovac et al. [10] describe the method as straightforward to apply and rapidly learn, even for surgeons with limited experience. However, it is of the utmost importance to maintain the correct distance (a minimum of 20 cm) and angle of incidence of the camera’s light beam in order to achieve optimal autofluorescence detection. In certain instances, the site of incision or the depth of the PG may impede the accurate visualization of the fluorescence. The wide variability of anatomical distribution by site and depth may contribute to the lack of identification, particularly for the lower PG [43]. The lack of glandular identification may be caused by the poor response of NIRAF for deep tissues beyond 3–5 mm [23,40,44]. In such circumstances, the surgeon is required to maneuver the device with due attention to the operating field and screen, in order to achieve optimal imaging results. Other studies corroborate relative ease of performing the procedure, particularly in comparison to alternative methods of intraoperative parathyroid identification [45]. It can thus be argued that NIRAF may be of significant assistance to experienced surgeons working in high-volume centres when operating on cases that are more complex, although as previously stated in such centres the incidence of POH is already lower, but especially to less-experienced surgeons working in non-high-volume centres.

The findings of our study indicate that the utilization of NIRAF results in the prolongation of unilateral surgical procedures, whereas bilateral procedures are conducted with greater expediency when NIRAF is employed. Nevertheless, no statistically significant difference was observed when compared to the respective non-NIRAF groups. Despite some studies in the literature indicating an increase in operating times associated with this method, they lack any quantification of surgical times [41]. Lerchenberg et al. reported a five- to ten-minute increase in average operating time when using the NIRAF technique [46]. This increase, combined with the high costs of purchasing AF instrumentation, may offset its advantages to a certain extent. Some authors posit that these constitute the primary obstacles to the routine implementation of the method in non-experimental clinical settings [47]. The differences reported in the length of the surgical procedures assisted by NIRAF may be attributed to the intraoperative installation of the system. Specifically, the assembly of the equipment, induction of AF, and the need to obtain a dark operating room appear to have a significant impact only in the unilateral procedure. This is because most of the steps described are already completed at the time of contralateral lobectomy, resulting in the reduction of the total time required to find the PG in bilateral procedures. A comparison between the time required for accurate visual identification of the PG and the overall NIRAF application time could confirm the increased time and operative cost associated with the procedure [41].

Finally, our analysis showed a correlation between postoperative LHS and postoperative complications. Consistently with literature, the two complications with the greatest impact on postoperative LHS were POH and hemorrhage [31]. Since the rates of postoperative hemorrhage had a low incidence in our case history, as well as in the literature, it follows that the main factor influencing the LHS is POH alone. However, despite the fact that patients undergoing NIRAF-assisted thyroid surgery were discharged earlier compared to those who underwent procedures without NIRAF, no clear impact of the use of the NIRAF method on postoperative hospitalization was found. This lack of statistical significance can be attributed to the small sample size and the reduced occurrence of hypocalcemia in this group. However, considering the significantly lower occurrence of POH after NIRAF-assisted procedures and the known role of POH in prolonging hospitalization, it may be argued that NIRAF clearly helps in reducing the LHS after surgery. Considering that the average cost per each day of surgical ward hospitalization accounts for approximately €200 to €400 but has been reported to reach approximately US $3000 [7], the indirect effect of NIRAF in reducing LHS by directly reducing POH would beneficially impact the NHS expenditure.

Our study has some potential limitations. First of all, its retrospective design. A possible temporal bias arises from the use of a historical non-contemporaneous control group. Nonetheless, despite the possible temporal bias, the members of the surgical equipe performing thyroid surgical procedures have remained the same over the years considered. The limited number of patients in the NIRAF group and the imbalance deriving from its size compared to the control group may affect the statistical power or the analyses.

Future randomized control trials or propensity score matching studies encompassing larger populations may provide further confirmation of our results and shed more light on the potential economic impact deriving from the better clinical outcomes. In an era of attention to healthcare spending, the intrinsic costs of NIRAF equipment could be offset by the gain associated with decreased rate of postoperative complications and shorter hospitalization. Although there is no available cost analysis comparing different operative methods, some authors are starting to highlight these economic advantages of modern AF techniques [41].

## 5. Conclusions

In the context of thyroid surgery, NIRAF represents a safe method with no documented risks to the patient. The procedure is relatively straightforward and does not significantly prolong the surgical time. Our results suggest that the systematic integration of NIRAF also within non-high-capacity centres may strongly contributes to a reduction in the incidence of POH after thyroid surgery by correctly identifying PGs intraoperatively. This, in turn, may decrease the postoperative LHS and healthcare expenditure. It is therefore conceivable that this method could be standardized and implemented within non-high-volume centres in the near future. The objective would be twofold: firstly, to enhance patient care outcomes; and secondly, to potentially contribute to a reduction in the overall cost of hospitalization for healthcare facilities.

## Figures and Tables

**Table 1:** Characteristics of patients. Group 1 with intraoperative NIRAF use; group 2 without use of NIRAF. [Results are reported +/− standard deviation (SD) when necessary. Fisher’s exact test (TEF) for categorical variables. Student’s *t*-test (TS) for continuous variables. Statistical significance at *p* < 0.05].

	Group 1 (n = 27)	Group 2 (n = 356)	*p* Value	Test
**Sex (Male:Female)**	7:20	158:198	0.0709	TEF
**Age (years, average ± SD)**	52.85 ± 17.24	53.66 ± 15.51	0.7951	TS
**BMI (Kg/m^2^, average ± SD)**	25.74 ± 4.16	26.00 ± 5.10	0.7971	TS
**Smoker**	3	58	0.5953	TEF
**Former smoker**	2	34	1	TEF
	**Preoperative diagnosis (n)**			
**Benign disease**	8	253	0.0145	TEF
**Malignant disease**	5	4	0.0001	TEF
**Suspect of malignancy**	14	99	0.0143	TEF
	**Type of surgery (n)**			
**Total thyroidectomy**	19	286	0.2191	TEF
**Hemithyroidectomy**	8	70	0.2191	TEF
	**Postoperative diagnosis (n)**			
**Benign disease**	18	288	0.0838	TEF
**Malignant disease**	8	67	0.2057	TEF
**Uncertain**	1	1	0.1362	TEF
**Length of hospital stay (days, average ± SD)**	4.33 ± 2	4.59 ± 4.46	0.7600	TS

**Table 2:** Number of potentially unidentified parathyroid glands (PGs) during the surgical procedure either by NIRAF (Group 1) or conventional techniques (Group 2), and number of PGs accidentally removed following non-identification. [Fisher’s exact test (TEF) for categorical variables. Statistical significance at *p* < 0.05].

	Group 1 (n = 27)	Group 2 (n = 356)	*p* Value	Test
**Unidentified PG**	3	22	0.4049	TEF
**Accidental PG removal**	0	20	0.3819	TEF

**Table 3:** Postoperative complications in Group 1 (intraoperative NIRAF) and Group 2 (without NIRAF). Results are the number of times the complication occurred in the selected group. [Fisher’s exact test (TEF) for categorical variables. Statistical significance at *p* < 0.05].

Complication	Group 1 (n = 27)	Group 2 (n = 356)	*p* Value	Test
**Transient hypocalcemia**	5 (18.5%)	163 (45.8%)	0.0078	TEF
**Permanent hypocalcemia**	0	4	1	TEF
**Loss of signal**	0	21	0.3833	TEF
**Permanent cord palsy**	0	1	1	TEF
**Hemorrhage**	0	12	1	TEF

**Table 4:** Postoperative complications of hemithyroidectomy procedures. Results are the number of times the complication occurred in the selected group. [Fisher’s exact test (TEF) for categorical variables. Statistical significance at *p* < 0.05].

Complication	Hemithyroidectomy with NIRAF (n = 8)	Hemithyroidectomy Without NIRAF (n = 70)	*p* Value	Test
**Transient hypocalcemia**	0	12	0.3457	TEF
**Permanent hypocalcemia**	0	0	1	TEF
**Loss of signal**	0	9	0.5859	TEF
**Permanent cord palsy**	0	1	1	TEF
**Hemorrhage**	0	0	1	TEF

**Table 5:** Postoperative complications of total thyroidectomy procedures. Results are the number of times the complication occurred in the selected group. [Fisher’s exact test (TEF) for categorical variables. Statistical significance at *p* < 0.05].

Complication	Total Thyroidectomy with NIRAF (n = 19)	Total Thyroidectomy Without NIRAF (n = 286)	*p* Value	Test
**Transient hypocalcemia**	5	151	0.0321	TEF
**Permanent hypocalcemia**	0	4	1	TEF
**Loss of signal**	0	12	1	TEF
**Permanent cord palsy**	0	0	1	TEF
**Hemorrhage**	0	12	1	TEF

**Table 6:** Correlation between major complications and postoperative length of stay. Comparison between all the patients who experienced a specific major complication versus the rest of the patients who did not experience it. [Results are reported +/− standard deviation (SD) when necessary. Student’s *t*-test (TS) for continuous variables. Statistical significance at *p* < 0.05].

Complication (Number of Patients Who Experienced It)	Average Length of Stay for Complicated Patients (Days)	Average Length of Stay for Non-Complicated Patients (Days)	*p* Value	Test
**Loss of signal (n = 21)**	5.95 ± 6.57	4.50 ± 4.17	0.1359	TS
**POH (n = 168)**	5.38 ± 5.56	3.94 ± 2.91	0.0012	TS
**Hemorrhage (n = 12)**	13.66 ± 17.47	4.28 ± 2.75	<0.0001	TS

**Table 7:** Correlation between complications and postoperative length of stay in Group 1 (intraoperative use of NIRAF). [Student’s *t*-test (TS) for continuous variables. Statistical significance at *p* < 0.05].

Complication	Average Length of Stay for Group 1 Complicated Patients (Days)	Average Length of Stay for Group 1 Non-Complicated Patients (Days)	*p* Value	Test
**Dysphonia (n = 0)**	-	4.33 ± 2	-	TS
**POH (n = 5)**	7 ± 2.96	3.6 ± 0.50	<0.0001	TS
**Hemorrhage (n = 0)**	-	4.33 ± 2	-	TS

**Table 8:** Correlation between complications and postoperative length of stay in Group 2 (without NIRAF). [Standard deviation (SD). Student’s *t*-test (TS) for continuous variables. Statistical significance at *p* < 0.05].

Complication	Average Length of Stay for Group 2 Complicated Patients (Days +/− SD)	Average Length of Stay for Group 2 Non-Complicated Patients (Days +/− SD)	*p* Value	Test
**Dysphonia (n = 21)**	5.95 ± 6.57	4.51 ± 4.30	0.1523	TS
**POH (n = 163)**	5.31 ± 5.61	3.99 ± 3.07	0.0049	TS
**Hemorrhage (n = 12)**	13.66 ± 17.47	4.28 ± 2.80	<0.0001	TS

**Table 9:** Operating time (minutes) and length of hospitalization (days) for hemithyroidectomy procedures. [Standard deviation (SD). Student’s *t*-test (TS) for continuous variables. Statistical significance at *p* < 0.05].

	Hemithyroidectomy with NIRAF (n = 8)	Hemithyroidectomy Without NIRAF (n = 70)	*p* Value	Test
**Operating time** **(min, average ± SD)**	65 ± 15	57.91 ± 21.94	0.3778	TS
**Hospitalization** **(days, average ± SD)**	3	4.28 ± 7.24	0.6192	TS

**Table 10:** Operating time (minutes) and length of hospitalization (days) for total thyroidectomy procedures. [Standard deviation (SD). Student’s *t*-test (TS) for continuous variables. Statistical significance at *p* < 0.05].

	Total Thyroidectomy with NIRAF (n = 19)	Total Thyroidectomy Without NIRAF (n = 286)	*p* Value	Test
**Operating time** **(min, average ± SD)**	79.47 ± 8.133	86.10 ± 28.35	0.3111	TS
**Hospitalization** **(days, average ± SD)**	4.89 ± 2.15	4.49 ± 2.95	0.5608	TS

**Table 11:** Correlation between individual characteristics and complications. [Fisher’s exact test (TEF) for categorical variables. Student’s *t*-test (TS) for continuous variables. Statistical significance at *p* < 0.05].

	Complicated (n = 181)	Not Complicated (n = 202)	*p* Value	Test
**Sex (Male:Female)**	83:98	82:120	0.3037	TEF
**Age (years, average ± SD)**	53.61 ± 15.39	52.72 ± 15.69	0.5952	TS
**BMI (Kg/m^2^, average ± DS)**	25.85 ± 5.04	26.09 ± 5.05	0.6482	TS
	**Preoperative diagnosis (n)**			
**Benign**	123	135	0.8280	TEF
**Malignant**	5	4	0.7406	TEF
**Uncertain**	53	63	0.7386	TEF
**Morbus Basedow**	24	24	0.7578	TEF
	**Postoperative diagnosis (n)**			
**Benign**	140	166	0.2526	TEF
**Malignant**	41	34	0.1584	TEF
**Uncertain**	0	2	0.5002	TEF

**Table 12:** Average values of postoperative serum calcium (mg/dL) and PTH (pg/mL) of Group 1 (NIRAF) and Group 2 (without NIRAF). [Standard deviation (SD). Student’s *t*-test (TS) for continuous variables. Statistical significance at *p* < 0.05].

	Group 1 (n = 27)	Group 2 (n = 356)	*p* Value	Test
**Calcemia 24 h** **(mg/dL, average ± SD)**	8.70 ± 0.55	8.58 ± 0.61	0.3321	TS
**Calcemia 48 h** **(mg/dL, average ± SD)**	8.80 ± 0.50	8.61 ± 0.58	0.1040	TS
**Calcemia one month** **(mg/dL, average ± SD)**	9.46 ± 0.65	9.12 ± 0.54	0.0019	TS
**Calcemia six months** **(mg/dL, average ± SD)**	9.24 ± 0.79	9.14 ± 0.40	0.2393	TS
**Parathormone** **(pg/mL, average ± SD)**	50.11 ± 39.43	39.56 ± 29.93	0.1050	TS

## Data Availability

The datasets generated during and/or analysed during the current study are not publicly available but are available from the corresponding author on reasonable request.

## Abbreviations

The following abbreviations are used in this manuscript:

AF	Autofluorescence
D1	Detection pre-thyroidectomy
D2	Detection post-
D3	Detection out: detection on the surgical thyroid specimen after resection
FU	Follow-up
IONM	Intraoperative neuromonitoring
IRB	Institutional Review Board
LHS	Length of hospitalization
LOS	Loss of signal
NIRAF	Near-infrared autofluorescence
PTH	Parathormone
PG	Parathyroid glands
POH	Postoperative hypocalcemia
RLN	Recurrent laryngeal nerve
SD	Standard deviation
TEF	Fisher’s exact test
TS	Student’s *t*-test

## References

[1] Tredici P., Grosso E., Gibelli B., Massaro M., Arrigoni C., Tradati N. (2011). Identification of patients at high risk for hypocalcemia after total thyroidectomy. Acta Otorhinolaryngol. Ital..

[2] Sitges-Serra A., Ruiz S., Girvent M., Manjón H., Dueñas J.P., Sancho J.J. (2010). Outcome of protracted hypoparathyroidism after total thyroidectomy. Br. J. Surg..

[3] Herranz González-Botas J., Lourido Piedrahita D. (2013). Hypocalcaemia after total thyroidectomy: Incidence, control and treatment. Acta Otorrinolaringol. Esp..

[4] Merchavy S., Forest V.-I., Marom T., Hier M.P., Mlynarek A.M., McHugh T., Payne R.J. (2014). Incidence of Postoperative Hypocalcemia following Total Thyroidectomy versus Completion Thyroidectomy. Otolaryngol.–Head Neck Surg..

[5] Bergenfelz A., Jansson S., Kristoffersson A., Mårtensson H., Reihnér E., Wallin G., Lausen I. (2008). Complications to thyroid surgery: Results as reported in a database from a multicenter audit comprising 3,660 patients. Langenbecks Arch. Surg..

[6] Edafe O., Antakia R., Laskar N., Uttley L., Balasubramanian S.P. (2014). Systematic review and meta-analysis of predictors of post-thyroidectomy hypocalcaemia. Br. J. Surg..

[7] Zahedi Niaki N., Singh H., Moubayed S.P., Leboeuf R., Tabet J.-C., Christopoulos A., Ayad T., Olivier M.-J., Guertin L., Bissada E. (2014). The cost of prolonged hospitalization due to postthyroidectomy hypocalcemia: A case-control study. Adv. Endocrinol..

[8] Paras C., Keller M., White L., Phay J., Mahadevan-Jansen A. (2011). Near-infrared autofluorescence for the detection of parathyroid glands. J. Biomed. Opt..

[9] Sehnem L., Noureldine S.I., Avci S., Isiktas G., Elshamy M., Saito Y., Ahmed A.H., Tierney H.T., Trinh L.N., Karcioglu A.S. (2023). A multicenter evaluation of near-infrared autofluorescence imaging of parathyroid glands in thyroid and parathyroid surgery. Surgery.

[10] Makovac P., Muradbegovic M., Mathieson T., Demarchi M.S., Triponez F. (2022). Preliminary experience with the EleVision IR system in detection of parathyroid glands autofluorescence and perfusion assessment with ICG. Front. Endocrinol..

[11] Solorzano C.C., Thomas G., Baregamian N., Mahadevan-Jansen A. (2020). Detecting the Near Infrared Autofluorescence of the Human Parathyroid Hype or Opportunity?. Ann. Surg..

[12] Paek S.H., Lee Y.M., Min S.Y., Kim S.W., Chung K.W., Youn Y.K. (2013). Risk factors of hypoparathyroidism following total thyroidectomy for thyroid cancer. World J. Surg..

[13] Stack B.C., Bimston D.N., Bodenner D.L., Brett E.M., Dralle H., Orloff L.A., Pallota J., Snyder S.K., Wong R.J., Randolph G.W. (2015). American association of clinical endocrinologists and American college of endocrinology disease state clinical review: Postoperative hypoparathyroidism-definitions and management. Endocr. Pract..

[14] Ozemir I.A., Buldanli M.Z., Yener O., Leblebici M., Eren T., Baysal H., Alimoglu O. (2016). Factors affecting postoperative hypocalcemia after thyroid surgery: Importance of incidental parathyroidectomy. North. Clin. Istanb..

[15] Nair C.G., Babu M.J., Menon R., Jacob P. (2013). Hypocalcaemia following total thyroidectomy: An analysis of 806 patients. Indian J. Endocrinol. Metab..

[16] Locati L., Crivellari G., Basolo F., Calo P.G., Cantisani V., Deandreis D., Del Rio P., Durante C., Furlani L., Ibrahim T. (2021). Linee Guida Tumori Della Tiroide, AIOM.

[17] Kim D.H., Lee S., Jung J., Kim S., Kim S.W., Hwang S.H. (2022). Near-infrared autofluorescence-based parathyroid glands identification in the thyroidectomy or parathyroidectomy: A systematic review and meta-analysis. Langenbecks Arch. Surg..

[18] Abbaci M., De Leeuw F., Breuskin I., Casiraghi O., Ben Lakhdar A., Ghanem W., Laplace-Builhé C., Hartl D. (2018). Parathyroid gland management using optical technologies during thyroidectomy or parathyroidectomy: A systematic review. Oral Oncol..

[19] De Leeuw F., Breuskin I., Abbaci M., Casiraghi O., Mirghani H., Ben Lakhdar A., Laplace-Builhé C., Hartl D. (2016). Intraoperative Near-infrared Imaging for Parathyroid Gland Identification by Auto-fluorescence: A Feasibility Study. World J. Surg..

[20] Kahramangil B., Dip F., Benmiloud F., Falco J., de La Fuente M., Verna S., Rosenthal R., Berber E. (2018). Detection of Parathyroid Autofluorescence Using Near-Infrared Imaging: A Multicenter Analysis of Concordance Between Different Surgeons. Ann. Surg. Oncol..

[21] Falco J., Dip F., Quadri P., de la Fuente M., Prunello M., Rosenthal R.J. (2017). Increased identification of parathyroid glands using near infrared light during thyroid and parathyroid surgery. Surg. Endosc..

[22] Wang B., Zhu C.R., Liu H., Yao X.-M., Wu J. (2021). The Accuracy of Near Infrared Autofluorescence in Identifying Parathyroid Gland During Thyroid and Parathyroid Surgery: A Meta-Analysis. Front. Endocrinol..

[23] Kim S.W., Lee H.S., Ahn Y.C., Park C.W., Jeon S.W., Kim C.H., Ko J.B., Oak C., Kim Y., Lee K.D. (2018). Near-Infrared Autofluorescence Image-Guided Parathyroid Gland Mapping in Thyroidectomy. J. Am. Coll. Surg..

[24] Kose E., Rudin A.V., Kahramangil B., Moore E., Aydin H., Donmez M., Krishnamurthy V., Siperstein A., Berber E. (2020). Autofluorescence imaging of parathyroid glands: An assessment of potential indications. Surgery.

[25] Takahashi T., Yamazaki K., Ota H., Shodo R., Ueki Y., Horii A. (2021). Near-Infrared Fluorescence Imaging in the Identification of Parathyroid Glands in Thyroidectomy. Laryngoscope.

[26] Ladurner R., Sommerey S., Arabi N.A., Hallfeldt K.K.J., Stepp H., Gallwas J.K.S. (2017). Intraoperative near-infrared autofluorescence imaging of parathyroid glands. Surg. Endosc..

[27] Wolf H.W., Grumbeck B., Runkel N. (2019). Intraoperative verification of parathyroid glands in primary and secondary hyperparathyroidism using near-infrared autofluorescence (IOPA). Updates Surg..

[28] Solórzano C.C., Thomas G., Berber E., Wang T.S., Randolph G.W., Duh Q.-Y., Triponez F. (2021). Current state of intraoperative use of near infrared fluorescence for parathyroid identification and preservation. Surgery.

[29] Tjahjono R., Nguyen K., Phung D., Riffat F., Palme C.E. (2021). Methods of identification of parathyroid glands in thyroid surgery: A literature review. ANZ J. Surg..

[30] Tjahjono R., Phung D., Elliott M.S., Riffat F., Palme C.E. (2023). The Utility of Near-Infrared Autofluorescence for Parathyroid Gland Identification During Thyroid Surgery: A Single-Center Experience. Indian J. Otolaryngol. Head Neck Surg..

[31] Nagel K., Hendricks A., Lenschow C., Meir M., Hahner S., Fassnacht M., Wiegering A., Germer C.-T., Schlegel N. (2022). Definition and diagnosis of postsurgical hypoparathyroidism after thyroid surgery: Meta-analysis. BJS Open.

[32] Ritter K., Elfenbein D., Schneider D.F., Chen H., Sippel R.S. (2015). Hypoparathyroidism after total thyroidectomy: Incidence and resolution. J. Surg. Res..

[33] Testini M., Gurrado A., Lissidini G., Nacchiero M. (2007). Hypoparathyroidism after total thyroidectomy. Minerva Chir..

[34] Rosato L., Avenia N., Bernante P., De Palma M., Gulino G., Nasi P.G., Pelizzo M.R., Pezzullo L. (2004). Complications of thyroid surgery: Analysis of a multicentric study on 14,934 patients operated on in Italy over 5 years. World J. Surg..

[35] Benmiloud F., Godiris-Petit G., Gras R., Gillot J.-C., Turrin N., Penaranda G., Noullet S., Chéreau N., Gaudart J., Chiche L. (2020). Association of Autofluorescence-Based Detection of the Parathyroid Glands During Total Thyroidectomy With Postoperative Hypocalcemia Risk: Results of the PARAFLUO Multicenter Randomized Clinical Trial. JAMA Surg..

[36] Benmiloud F., Rebaudet S., Varoquaux A., Penaranda G., Bannier M., Denizot A. (2018). Impact of autofluorescence-based identification of parathyroids during total thyroidectomy on postoperative hypocalcemia: A before and after controlled study. Surgery.

[37] Dip F., Falco J., Verna S., Prunello M., Loccisano M., Quadri P., White K., Rosenthal R. (2019). Randomized Controlled Trial Comparing White Light with Near-Infrared Autofluorescence for Parathyroid Gland Identification During Total Thyroidectomy. J. Am. Coll. Surg..

[38] Van Slycke S., Van DenHeede K., Brusselaers N., Vermeersch H. (2021). Feasibility of Autofluorescence for Parathyroid Glands During Thyroid Surgery and the Risk of Hypocalcemia: First Results in Belgium and Review of the Literature. Surg. Innov..

[39] Huang J., He Y., Wang Y., Chen X., Zhang Y., Chen X., Huang Z., Fang J., Zhong Q. (2023). Prevention of hypoparathyroidism: A step-by-step near-infrared autofluorescence parathyroid identification method. Front. Endocrinol..

[40] Barbieri D., Indelicato P., Vinciguerra A., Di Marco F., Formenti A.M., Trimarchi M., Bussi M. (2021). Autofluorescence and Indocyanine Green in Thyroid Surgery: A Systematic Review and Meta-Analysis. Laryngoscope.

[41] Weng Y.J., Jiang J., Min L., Ai Q., Chen D., Chen W., Huang Z. (2021). Intraoperative near-infrared autofluorescence imaging for hypocalcemia risk reduction after total thyroidectomy: Evidence from a meta analysis. Head Neck..

[42] Prete F.P., Panzera P.C., Di Meo G., Pasculli A., Sgaramella L.I., Calculli G., Dimonte R., Ferrarese F., Testini M., Gurrado A. (2022). Risk factors for difficult thyroidectomy and postoperative morbidity do not match: Retrospective study from an endocrine surgery academic referral centre. Updates Surg..

[43] Santini L., Castillo L., Poissonnet G. (2001). Chirurgia Delle Ghiandole Paratiroidi.

[44] Ladurner R., Al Arabi N., Guendogar U., Hallfeldt K., Stepp H., Gallwas J. (2018). Near-infrared autofluorescence imaging to detect parathyroid glands in thyroid surgery. Ann. R. Coll. Surg. Engl..

[45] Qian B., Zhang X., Bing K., Hu L., Qu X., Huang T., Shi W., Zhang S. (2022). Real-time intraoperative near-infrared autofluorescence imaging to locate the parathyroid glands: A preliminary report. Biosci. Trends.

[46] Lerchenberger M., Al Arabi N., Gallwas J.K.S., Stepp H., Hallfeldt K.K.J., Ladurner R. (2019). Intraoperative Near-Infrared Autofluorescence and Indocyanine Green Imaging to Identify Parathyroid Glands: A Comparison. Int. J. Endocrinol..

[47] Di Marco A.N., Palazz F.F. (2020). Near-infrared autofluorescence in thyroid and parathyroid surgery. Gland Surg..

